# Metastatic neuroblastoma cancer stem cells exhibit flexible plasticity and adaptive stemness signaling

**DOI:** 10.1186/s13287-015-0002-8

**Published:** 2015-02-20

**Authors:** Vijayabaskar Pandian, Satishkumar Ramraj, Faizan H Khan, Tasfia Azim, Natarajan Aravindan

**Affiliations:** Department of Radiation Oncology, University of Oklahoma Health Sciences Center, 940 Stanton L. Young Blvd., BMSB 737, Oklahoma City, OK 73104 USA

## Abstract

**Introduction:**

High-risk neuroblastoma (HR-NB) presenting with hematogenous metastasis is one of the most difficult cancers to cure. Patient survival is poor. Aggressive tumors contain populations of rapidly proliferating clonogens that exhibit stem cell properties, cancer stem cells (CSCs). Conceptually, CSCs that evade intensive multimodal therapy dictate tumor progression, relapse/recurrence, and poor clinical outcomes. Herein, we investigated the plasticity and stem-cell related molecular response of aggressive metastatic neuroblastoma cells that fit the CSC model.

**Methods:**

Well-characterized clones of metastatic site-derived aggressive cells (MSDACs) from a manifold of metastatic tumors of clinically translatable HR-NB were characterized for their CSC fit by examining epithelial-to-mesenchymal transition (EMT) (E-cadherin, N-Cadherin), survival (NFκB P65, p50, IκB and pIκB) and drug resistance (ABCG2) by immunoblotting; pluripotency maintenance (Nanog, SOX2) by immunofluorescence; and EMT and stemness related transcription of 93 genes by QPCR profiling. Plasticity of MSDACs under sequential alternation of culture conditions with serum and serum-free stem-cell conditions was assessed by clonal expansion (BrdU incorporation), tumorosphere formation (anchorage independent growth), EMT and stemness related transcriptome (QPCR profiling) and validated with MYC, SOX2, EGFR, NOTCH1 and CXCL2 immunoblotting.

**Results:**

HR-NB MSDACs maintained in alternated culture conditions, serum-free stem cell medium to growth medium with serum and vice versa identified its flexible revocable plasticity characteristics. We observed signatures of stem cell-related molecular responses consistent with phenotypic conversions. Successive reintroduction to the favorable niche not only regained identical EMT, self-renewal capacity, pluripotency maintenance, and other stem cell-related signaling events, but also instigated additional events depicting aggressive adaptive plasticity.

**Conclusions:**

Together, these results demonstrated the flexible plasticity of HR-NB MSDACs that typically fit the CSC model, and further identified the intrinsic adaptiveness of the successive phenotype switching that clarifies the heterogeneity of HR-NB. Moreover, the continuous ongoing acquisition of stem cell-related molecular rearrangements may hold the key to the switch from favorable disease to HR-NB.

**Electronic supplementary material:**

The online version of this article (doi:10.1186/s13287-015-0002-8) contains supplementary material, which is available to authorized users.

## Introduction

Neuroblastoma (NB), an extracranial solid tumor that arises from neural crest components of the sympathetic nervous system, is the most common cancer of infancy [1,2]. Although neural crest cells undergo progressive differentiation, there are subsets without differentiation under different lineages. These subsets are maintained within niches which could facilitate cell-fate changes when necessary, underscoring the developmental plasticity of this population [3,4]. The prognostic significance of the cellular heterogeneity of neural crest lineage cells in NB has begun to be described [5,6]. Clinical evidence has recognized cell morphology diversity with the presence of a variety of neural crest cell types in neuroblastoma including neuroblasts, melanocytes, glial cells and chondrocytes [7,8]. Clonal sublines from such neural crest cells identified three distinct types including: (1) small, rounded, loosely adherent cells with neurite-like processes, ‘N’ type cells; (2) large, flat, epithelial or fibroblast-like and highly substrate adherent cells, ‘S’ type; and (3) cells with intermediate morphology between ‘N’ and ‘S’ type cells, moderately substrate adherent and having small numbers of neurite-like processes, ‘I’ type. Further, studies have defined that both N and S type cells descended from a common precursor cell, and are capable of spontaneous bidirectional inter-conversion, ‘trans-differentiation,’ which is a prevalent phenomenon among human neuroblastoma cell lines. More importantly, studies have suggested I-type cells could represent a cellular intermediate in the trans-differentiation process, and the phenotypic conversion could be regulated by extrinsic and/or intrinsic factors.

Clinically, a higher percentage of I-type cells associated with augmented tumorigenicity as well as increased rates of tumor relapse [9]. Interestingly, these cells expressed CD133 and showed asymmetric cell division [9,10]. Other studies revealed that NB cells express neural precursor markers, including CD34, ABCG2 and nestin [11-13]. Sixty-five percent of primary NB samples have side populations, providing further evidence that NB is a stem cell tumor [11]. Clinical and laboratory evidence suggests that several common human cancers contain populations of rapidly proliferating clonogens that can have a substantial impact on tumor control following therapy [14]. For many cancers, including NB, it has been hypothesized that the tumor cells responsible for failures in long-term remission exhibit stem cell properties [15-21]. Since more than half of the patients with high-risk NB will relapse with hematogenous metastasis [22] despite intensive multimodal therapy [23-32], we investigated the plasticity of stem-like aggressive NB cells. Plasticity is the capability of a tumor cell to adapt to its microenvironment and alter its phenotype.

Adult neural crest-derived cells have been shown to retain stem cell properties [33]. Studies have consistently demonstrated that such neural crest stem cell (NCSC) populations often mimic transcription expression profiles of both embryonic stem cells and early neural crest cells [34-36]. Recent breakthrough investigations recognized the generation of induced pluripotent stem cells (iPSCs) [34,36] and showed that iPSCs can be derived by the manipulation of selective transcription factors. Given high-risk NB’s heterogeneity, vigorous progression, and therapy resistance, we hypothesize that selective ‘to-and-from’ acquisition of genetic/molecular rearrangements pertaining to the epithelial-to-mesenchymal transition (EMT), pluripotency maintenance, self-renewal capacity, and drug resistance may facilitate the better survival of such aggressive clones. Studies have shown the progression of solid tumors from a minor population of cancer stem cells (CSCs) with altered expression of selective molecules, self-renewal capacity, and differentiation [37]. These types of tumor cell subpopulations with altered stem-like phenotypes have been identified in several tumor systems, including leukemia, breast, brain and colon cancers [38-41]. Neuroblastoma CSCs that exhibit self-renewal and dynamic proliferation were also capable of generating non-adherent tumorospheres with enriched stemness [42]. Accordingly, we investigated the ongoing continuous modifications in the stem cell-related transcriptional machinery of aggressive metastatic NB cells.

This study employed the derived clones of CSCs from a manifold of metastatic tumors of a unique, validated, and clinically translatable mouse model of high-risk aggressive NB. Distinctively, the current study demonstrated the potential plasticity of derived aggressive clones in sequentially alternated microenvironments *ex vivo.* Further, a comprehensive, quantitative examination of stemness-associated transcriptional machinery showed heightened transcription of EMT-, pluripotency maintenance-, self-renewal capacity-, and drug resistance-related molecules, and identified plasticity-associated subsets of regained, lost, or activated stem cell-related molecules, signifying the plasticity and corresponding functional stem cell-related molecular rearrangements in high-risk aggressive neuroblastoma.

## Methods

### Cell culture

**Figure 1:**
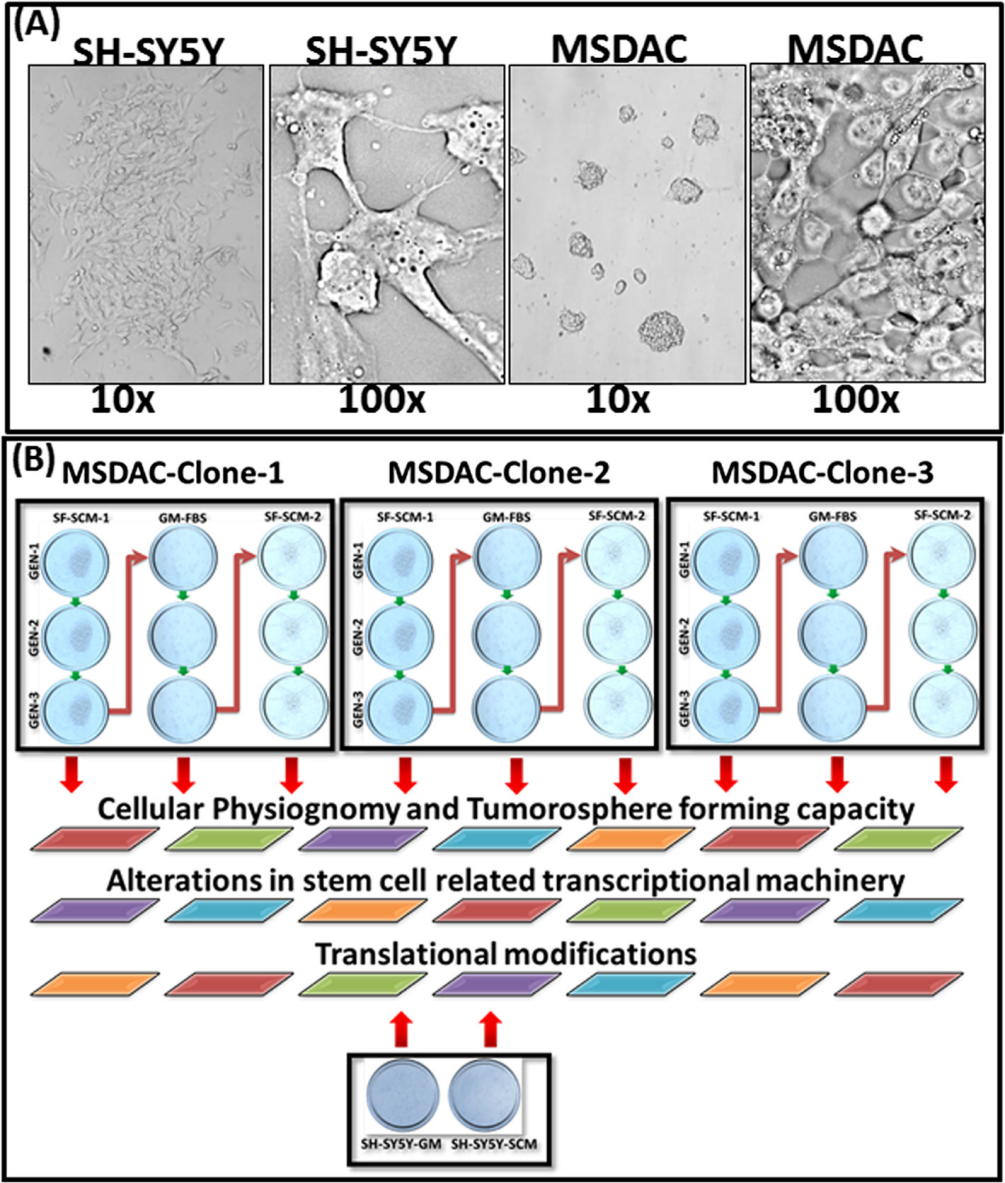
**Morphometrics of the parental SH-SY5Y and metastatic site derived aggressive cells and the schematic representation of the experimental workflow. (A)** Representative light microscope photographs of parental SH-SY5Y cells and metastatic site-derived aggressive cells (MSDACs) maintained in serum-free stem cell medium. Parental cells exhibited monolayer spreading with organized neurites (100 ×). MSDACs are more spherical, smaller in size (100 ×), and formed organized tumorospheres (10 ×). **(B)** Schematic representation of experimental workflow: Three well characterized individual clones of MSDACs were alternated between stabilized (three generations) microenvironment simulations with serum-free stem cell medium to growth medium with serum and reintroduced back to serum-free stem cell medium. Parental cells grown in routine medium and serum-free stem cell medium were included as controls. Cancer stem cell physiognomy, tumorosphere formation capacity, and transcriptional and translational rearrangements associated with phenotype conversions were assessed at the end of each phase and in controls.

The parental human NB (SH-SY5Y) cell line obtained from ATCC (Manassas, VA, USA) was cultured and maintained as described earlier [43]. We maintained metastatic site-derived aggressive cell (MSDACs, Figure 1A) clones that were established from the manifold of metastatic tumors of a clinically mimicking animal model of human high-risk metastatic disease *ex vivo* in serum-free stem cell medium DMEM:F12 with 1% N2 supplement, 2% B27 supplement, 20 ng/ml platelet-derived growth factor (hPDGF), 100 ng/ml epidermal growth factor (EGF), and 1% antibiotic-antimycotic). High-risk aggressive neuroblastoma animal model development and the derivation of the MSDAC clones from multiple metastatic tumors were reported elsewhere. MSDACs were sequentially characterized with karyotyping, whole genome array-Comparative Genome Hybridization (CGH) analysis, whole genome gene expression, translational expression of tumor progression-related proteins, miRNA profiling, tumorosphere-forming capacity, and stemness (unpublished data). In this study, early passages of derived MSDACs were examined for their plasticity and associated stemness-related transcriptional/translational alterations.

### Cellular plasticity experiments

To determine the potential of aggressive MSDACs in best-fit-survival in altered growing conditions and to underscore its microenvironment-driven plasticity, we examined their growth patterns and tumorosphere-forming capacity *ex vivo.* A schematic representation of the experimental workflow is presented in Figure 1B. Briefly, isolated and characterized clones of MSDACs from multiple metastatic tumors were maintained in serum-free stem cell medium (SF-SCM-1) for three generations. At the end of the third generation, the cells were transferred to the parental cell-culture conditions, that comprises serum supplemented DMEM:F12 medium (growth medium with fetal bovine serum (GM-FBS)) without growth factors. Cells maintained in GM-FBS for three generations were then re-introduced to serum-free stem cell medium (SF-SCM-2). Parallel primary SF-SCM-1 and GM-FBS controls and phase-fixed SF-SCM-1 → GM-FBS controls were also included. Parental SH-SY5Y cells grown in serum-free stem cell medium served as a baseline control. All experiments were repeated in triplicate utilizing alternate clones of MSDACs. Cell loss and spreading, and formation of well-organized tumorospheres, was assessed with phase contrast light microscopy. In parallel, 1,000 cells plated in 96-well culture plates were examined with high content real-time fluorescent imaging. Cells were stained with DiI (1,1′-dioctadecyl-3,3,3′,3′-tetramethylindocarbocyanine perchlorate (‘DiI’; DiIC18(3)), Life Technologies, Grand Island, NY, USA), an orange-red-fluorescent dye that is a long-term tracer for neuronal cells. Cells were imaged in real-time once every 20 minutes for an extended period of 18 hours using Operetta (Perkin Elmer, Inc., Waltham, MA, USA). Sequential images were reconstructed in Harmony (Perkin Elmer) to obtain a time-lapse video. Cells were also harvested under each experimental condition for subsequent endpoint analysis, including transcriptional QPCR profiling, immunoblotting, and high content quantitative immunofluorescence.

### Quantitative EMT and stemness-related transcriptome profiling

Total RNA extraction and real-time QPCR profiling were performed as described earlier [44,45]. We used custom-made transcriptome profilers (Realtimeprimers.com) pertaining to stemness and EMT signaling. We archived a unique stem cell-related gene profile (Table S1 in Additional file 1) and constructed the QPCR profiler in collaboration with realtimeprimers.com. We used this highly selected QPCR profiler instead of an all-encompassing gene array because the selected genes provide a well-characterized profile governing EMT, pluripotency maintenance, self-renewal capacity, and drug resistance that direct the plasticity of these aggressive cells. The ΔΔ^ct^ values calculated by normalizing the gene expression levels to that of housekeeping genes were then compared between groups. The relative expression level of each gene was expressed as fold change. Group-wise comparisons were made using t-test (for comparing SH-SY5Y versus MSDACs) and two-way analysis of variance (ANOVA) with Tukey’s *post-hoc* correction for comparing the gene loss, gain or regain in MSDACs utilizing GraphPad PRISM.

### Immunoblotting

Total protein extraction and immunoblotting were performed as described in our earlier studies [43,46]. In this study, the protein-transferred membranes were incubated with rabbit polyclonal anti-MYC, anti-Sox-2(Santa Cruz Biotechnology Inc., Santa Cruz, CA, USA), anti-ABGC2, and anti-nCadherin (Aviva Systems Biology Corp., San Diego, CA, USA) antibodies, and mouse monoclonal anti-NFκB-p65, anti-NFκB-p50, anti-eCadherin (Santa Cruz), anti-NOTCH-1 (Pierce Biotechnology, Rockford, IL, USA), and mouse polyclonal CXCL12 (eBioscience Inc., San Diego, CA, USA). Membranes were developed with the appropriate anti-mouse/anti-rabbit (BioRad Laboratories, Hercules, CA, USA) secondary antibody. Blots were stripped and reblotted with rabbit polyclonal anti-β-actin antibody (Gentex Inc., Irvine, CA, USA) or anti-α-tubulin to determine equal loading of the samples. Densitometry analysis was performed using Quantity One gel analysis software (BioRad). α-tubulin or β-actin normalized values are compared between groups using t-test or two way ANOVA with Bonferoni’s *post hoc* correction (GraphPad Prism) and a *P* value of less than one is considered statistically significant.

### Bromodeoxyuridine-incorporation assay

SH-SY5Y cells and the MSDACs grown in SF-SCM-1(3G), SF-SCM-1(3G) → GM-FBS(3G), or SF-SCM-1(3G) → GM-FBS(3G) → SCM-2(3G) were treated with bromodeoxyuridine (BrdU) (1 μM) for 2 hours, washed and fixed in sucrose supplemented 3% paraformaldehyde in PBS. Fixed cells were serially treated with 0.1% triton buffer and 2 M HCl with excessive intermittent PBS washing. The cells were then blocked (0.1% BSA in PBS), tagged with anti-BrdU mouse antibody (for 1 hour at 37°C), washed and second labelled with goat-anti mouse Alexa Fluor-488. Unlabeled nuclei were then stained with 4',6-diamidino-2-phenylindole (DAPI) and analyzed in Operetta high content confocal imaging. Experiments are repeated three times and for each well 25 different fields in 14 different Z planes were captured. Quantification of the BrdU incorporated nuclei are counted using Operetta integrated Columbus software.

### Anchorage-independent soft agar assay

MSDACs grown in SF-SCM-1(3G), SF-SCM-1(3G) → GM-FBS(3G), or SF-SCM-1(3G) → GM-FBS(3G) → SCM-2(3G) were suspended in defined medium containing 0.3% agar and seeded into 24-well culture plates. To avoid cell attachment, culture plates were pre-coated with 0.5% agar before cell seeding. Cultures were fed with fresh medium every three to five days and cultured for fourteen days. Colonies developed were fixed with 3.7% paraformaldehyde and stained with 0.1% crystal violet and imaged with the Vista-Vision Inverted trinocular microscope equipped with Moticam 5MP CMOS camera.

### High content confocal immunocytofluorescence

We examined the cellular localization and expression levels of SOX2 and Nanog in parental SH-SY5Y and in multiple clones of MSDACs using Operetta (Perkin Elmer) high content and quantitative confocal imaging. Paraformaldehyde-fixed SH-SY5Y cells and MSDACs were permeabilized (0.25% Triton X-100), blocked (1% BSA in PBS), and labelled with rabbit polyclonal anti-SOX2 and anti-nanog antibody (1: 200, Santa Cruz). Then, they were tagged with Alexa Fluor 488 fluorochrome conjugated anti-mouse secondary antibodies (Abcam). The nucleus was counter-labeled with DAPI. After washing, the plates were analyzed in Operetta, at least eight fields/well and three wells/clone, with a minimum of 21 Z planes. Unstained controls were included for both cell lines. Columbus software (Perkin Elmer) was used for quantitative image analysis.

## Results

### MSDACs from high-risk metastatic disease exhibited heightened stemness and fit the neuroblastoma cancer stem cell profile

**Figure 2:**
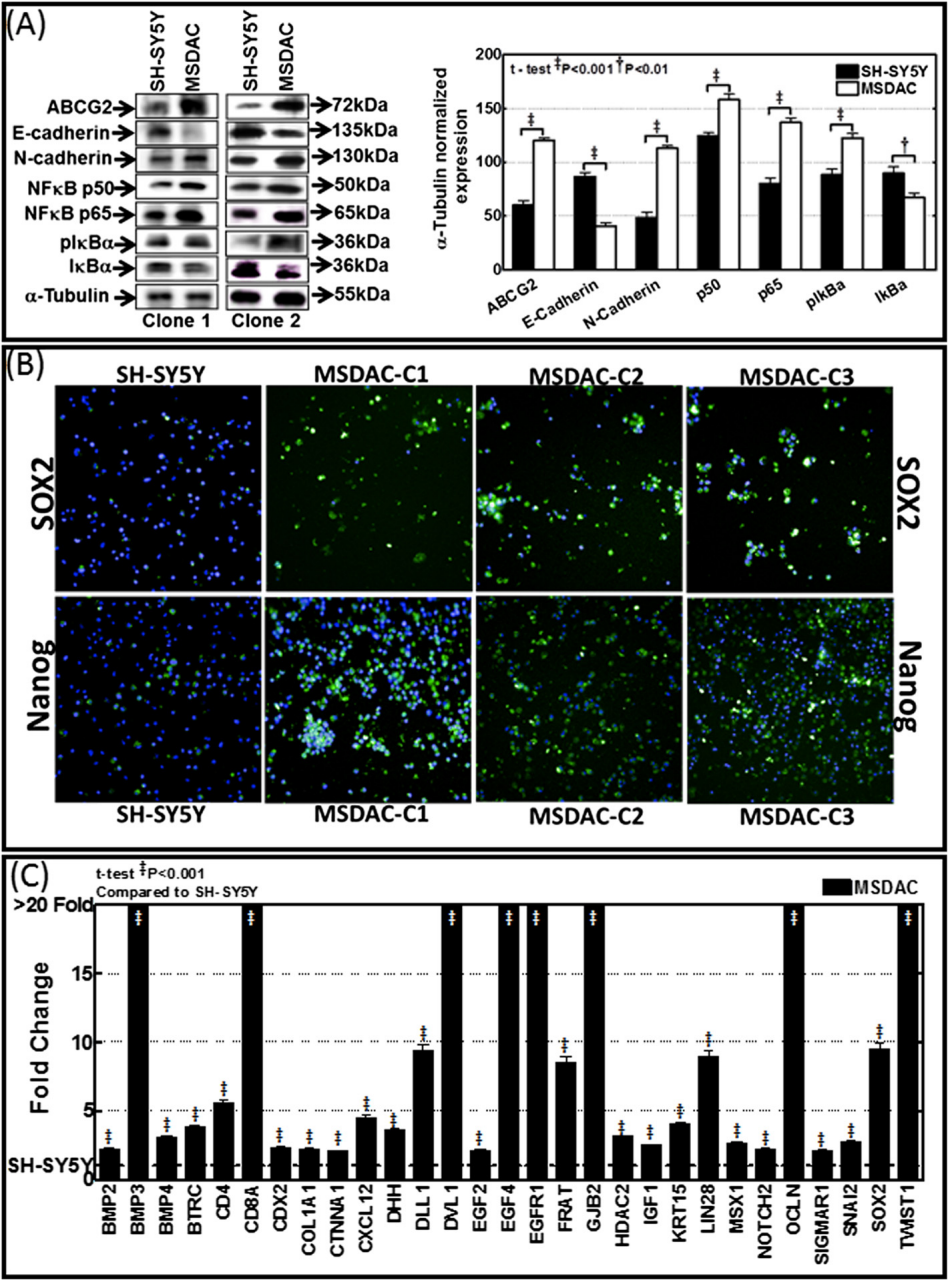
**Cancer stem cell characterization of MSDACs. (A)** Immunoblotting analysis showing activated EMT (increased N-cadherin and decreased E-cadherin), drug resistance (increased ABCG2), and survival response (increased p65/p50 and pIκBα) in MSDACs compared with parental SH-SY5Y cells. **(B)** High content confocal immunofluorescence showing increased expression levels of pluripotency maintenance factors SOX2 and NANOG in three different clones of MSDACs. **(C)** Results of QPCR profiling analysis showing transcriptional activation of 29 stem cell-related molecules in MSDACs maintained in serum-free stem cell medium, compared with SH-SY5Y cells. EMT, epithelial-to-mesenchymal transition; MSDACs, metastatic site-derived aggressive cells.

All MSDACs clones exhibited consistent uncontrolled growth in SF-SCM. Compared with the parental SH-SY5Y cells that have structured neurites, MSDACs are smaller in size and readily form organized tumorospheres (Figure 1A). Immunoblot analysis and high content confocal immunofluorescence (IF) revealed activated stem cell characteristics and cell survival signaling in MSDACs (Figure 2). We observed a robust activation of the EMT marker N-cadherin in MSDACs compared with parental SH-SY5Y cells. E-Cadherin was significantly (*P* <0.001) reduced in MSDACs, demonstrating an active EMT (Figure 1A). Likewise, we observed a significant (*P* <0.001) increase in the expression of the drug-resistance molecule AGBCG2 in MSDACs. Immunoblotting revealed a profound increase in the expression of the transcriptional switch NFκB that promotes cell survival. We observed a significant increase in the expression of NFκB-p65 and p50 in MSDACs, the heterodimeric formation of which functions as a transcriptional regulator (Figure 2A). Consistently, we observed a definite increase in IκB phosphorylation, indicating the release of NFκB from the cytoplasm. We found no alterations in the constitutive levels of IκB (Figure 2a). Moreover, the results of the high content IF analysis showed a strong pluripotency maintenance activity in MSDACs. We observed a significant increase in the expression levels of pluripotency-maintaining SOX2 and NANOG in various clones of MSDACs (Figure 2B), compared with the parental SH-SY5Y cells. Further, comprehensive EMT and stem cell-related quantitative transcriptional profiling revealed significant increases in the transcription of 29 stem cell-related molecules in MSDACs: BMP2, BMP3, BMP4, BTRC, CD4, CD8A, CDX2, COL1A1, CTNNA1, CXCL12, DHH, DLL1, DVL1, EGF2, EGF4, EGFR1, FRAT, GJB2, HDAC2, IGF1, KRT15, LIN28, MSX1, NOTCH2, OCLN, SIGMAR1, SNAI2, SOX2, and TWIST1 (Figure 2C). Parental SH-SY5y cells did not display increased transcription of these molecules. Together, these results suggest that the derived clones of MSDACs possess activated machinery that drives cell survival, EMT, drug resistance, and pluripotency maintenance.

### MSDACs exerted high levels of cellular plasticity

**Figure 3:**
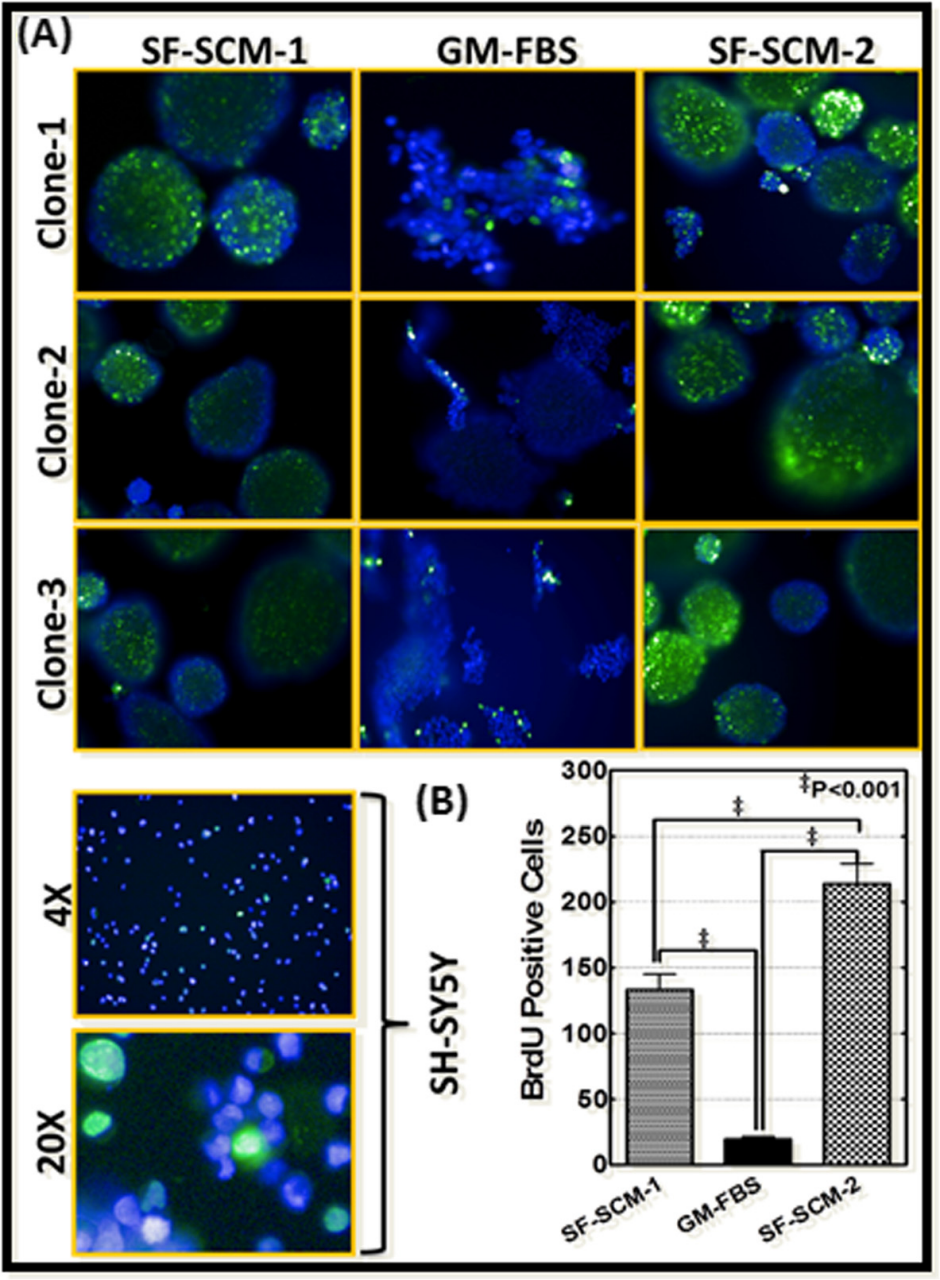
**Modulations in cell proliferation levels of MSDACs under alternated culture conditions. A)** Representative microphotographs of random fields obtained from Operetta high-content confocal imaging showing BrdU incorporation levels in three different clones of MSDACs maintained in serum-free stem cell medium for three generations: SF-SCM-1(3G), SF-SCM-1(3G) cells transferred and maintained in growth medium with FBS for three generations (SF-SCM-1(3G) → GM-FBS(3G), or the MSDACs reintroduced in SF-SCM for three generations (SF-SCM-1(3G) → GM-FBS(3G) → SF-SCM-2(3G). Parental SH-SY5Y cells maintained in growth medium are used as controls. **B)** Histograms obtained from Columbus image analysis showing significant decline in BrdU positive cells in SF-SCM-1(3G) → GM-FBS(3G) MSDACs and regained and amplified cell proliferation in MSDACs reintroduced in SF-SCM (SF-SCM-1(3G) → GM-FBS(3G) → SF-SCM-2(3G). Brd-U, bromodeoxyuridine; MSDACs, metastatic site-derived aggressive cells.

To underscore the plasticity of the MSDACs, we adopted sequential alternations of the culture conditions. A schematic representation of the experimental work flow is provided in Figure 1B. Parallel populations from three different MSDAC clones were examined for cell growth physiognomies, including proliferation, spreading or aggregation, and tumorosphere formation under different culture conditions. To further highlight cellular plasticity and avoid the possibility of delayed cell response inference, we maintained the populations for at least three generations under each culture condition. BrdU incorporation assay revealed a baseline proliferation in parental SH-SY5Y cells (Figure 3A lower panel). MSDACs (SF-SCM-1) on the other hand, showed an increased proliferation rate. Conversely, the proliferation rate was much slower in SF-SCM-1(3G) → GM-FBS(3G) MSDACs (Figure 3A). Notably, MSDACs reintroduced into the stem cell medium (SF-SCM-1(3G) → GM-FBS(3G) → SCM-2(3G)) demonstrated not only the regaining of cell proliferation, but also exhibited a heightened cell proliferation level (Figure 3A). Quantification of the BrdU incorporated nuclei recognized: significant (*P* <0.001) decrease in cell proliferation when MSDACs are introduced to GM-FBS; robust (*P* <0.001) increase when these cells are reintroduced to SF-SCM; and relatively amplified (*P* <0.001) cell proliferation in SF-SCM-2 as that of SF-SCM-1 (Figure 3B).

**Figure 4:**
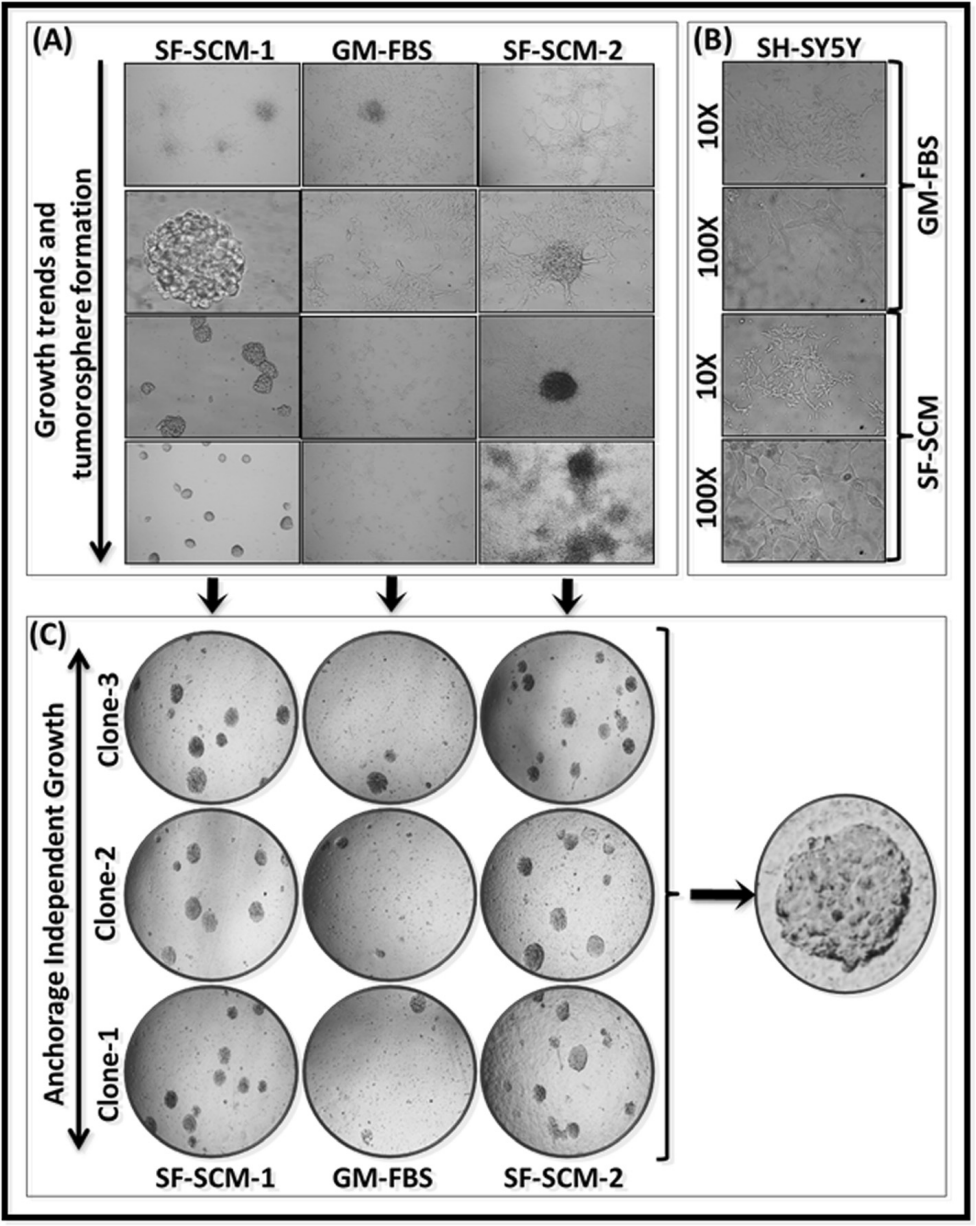
**Tumorosphere formation and anchorage independent growth capabilities of MSDACs in alternated culture conditions. (A)** Representative microphotographs showing growth trends, monolayer differentiation or cell aggregation, tumorosphere formation, and phenotype conversions in three different clones of MSDACs maintained in serum-free stem cell medium for three generations: SF-SCM-1(3G), SF-SCM-1(3G) cells transferred and maintained in growth medium with FBS for three generations (SF-SCM-1(3G) → GM-FBS(3G), or the MSDACs reintroduced in SF-SCM for three generations (SF-SCM-1(3G) → GM-FBS(3G) → SF-SCM-2(3G). **(B)** Parental SH-SY5Y cells maintained in growth medium or SF-SCM are used as controls. **(C)** Representative microphotographs of soft agar colony forming assay showing anchorage-independent growth signatures of MSDACs maintained in serum-free stem cell medium for three generations, transferred and maintained in growth medium with FBS for three generations or reintroduced in SF-SCM for three generations. FBS, fetal bovine serum; MSDACs, metastatic site-derived aggressive cells; SF-SCM, serum-free stem cell medium.

Parental SH-SY5Y cells grown in routine medium showed uniform monolayer spreading with no cellular aggregation or tumorosphere formation (Figure 4). Likewise, SH-SY5Y cells maintained in SF-SCM demonstrated monolayer spreading without tumorosphere formation. Cell spreading was relatively slower in such conditions. A few populations showed initial cell aggregation, but did not form any organized tumorospheres (Figure 4B). However, MSDACs grown in SF-SCM consistently formed organized tumorospheres across generations. The cell proliferation rate was evidenced by the increased size and number of tumorospheres (Figure 4A). A known number of cells from the third generation were stained with DiI and observed in real-time for a period of 18 hours. Staining revealed tumorosphere formation without monolayer cell spreading (Figure S1 in Additional file 2). Single cell suspensions obtained from tumorospheres of SF-SCM-1(3G) that were transferred to GM-FBS culture conditions exhibited parental cell-like monolayer spreading without tumorosphere formation. Consistent with the SH-SY5Y cells, these populations showed an initial cell aggregation, but formed no tumorospheres (Figure 4A). High content sequential observations of these populations (SF-SCM-1(3G) → GM-FBS(3G)) revealed no tumorosphere formation and further demonstrated a monolayer differentiation (Figure S2 in Additional file 3). Interestingly, SF-SCM-1(3G) → GM-FBS(3G) cells reintroduced to serum-free stem cell medium (SF-SCM-2) showed robust and organized tumorosphere formation despite their demonstrated monolayer differentiation in SF-SCM-1(3G) → GM-FBS (Figure 4A). Notably, these MSDACs (SF-SCM-1(3G) → GM-FBS(3G) → SCM-2(3G)) are more aggressive, as evidenced by the formation of more and larger tumorospheres (Figure S3 in Additional file 4).

Further, anchorage-independent cell growth assay exhibited marked numbers of defined colonies with SF-SCM-1(3G) MSDACs (Figure 4C). Evidently, soft-agar colony formation capability was completely reduced in SF-SCM-1(3G) → GM-FBS(3G) MSDACs. Conversely, MSDACSs reintroduced into the SF-SCM-2 (SF-SCM-1(3G) → GM-FBS(3G) → SCM-2(3G)) profoundly exhibited colony formation in soft agar. Relatively, the colonies formed by the cells from SF-SCM-2 were morphometrically bigger in a given period of time (Figure 4C). These phase specific anchorage-independent cell growth signatures corroborated well with the tumorosphere formation as well as cell proliferation data. Taken together, these results demonstrate that the human neuroblastoma MSDACs derived from a manifold of metastatic tumors are extremely plastic, depending on the environmental influence, and further show that these MSDACs can aggressively regain tumorigenic capacity given an appropriate niche .

### Plasticity and aggression of human neuroblastoma MSDACs substantiates altered stemness machinery

**Figure 5:**
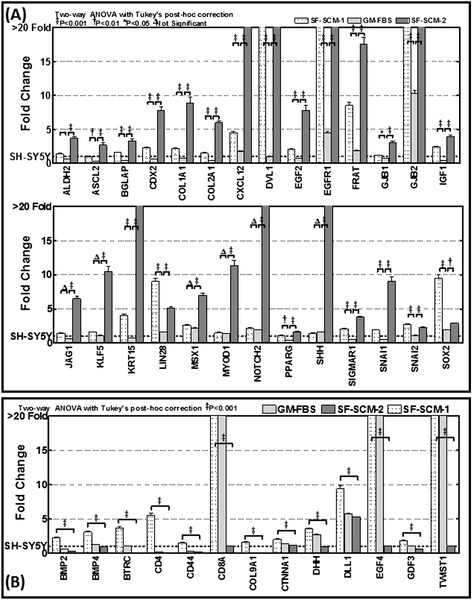
**QPCR profiling identifies regained and/or lost transcription of EMT and stem cell molecules in MSDACs cultured under alternated growth conditions. (A)** Histograms of QPCR profiling analysis showing stem cell-related transcriptional responses regained with the reverted CSC phenotype when MSDACs are reintroduced into SF-SCM. Interestingly, the rescue of stem cell transcriptional responses is relatively heightened when compared with the earlier CSC phenotype stage. **(B)** Histograms showing the panel of stem cell-related molecules that were significantly lost in the second phase CSC phenotype. Almost all of these molecules showed decreased expression in the non-favorable niche (GM-FBS) and never regained expression when returned to a favorable environment. CSC, cancer stem cell; EMT, epithelial-to-mesenchymal transition; GM-FBS, growth medium with fetal bovine serum; MSDACs, metastatic site-derived aggressive cells; SF-SCM, serum-free stem cell medium.

**Figure 6:**
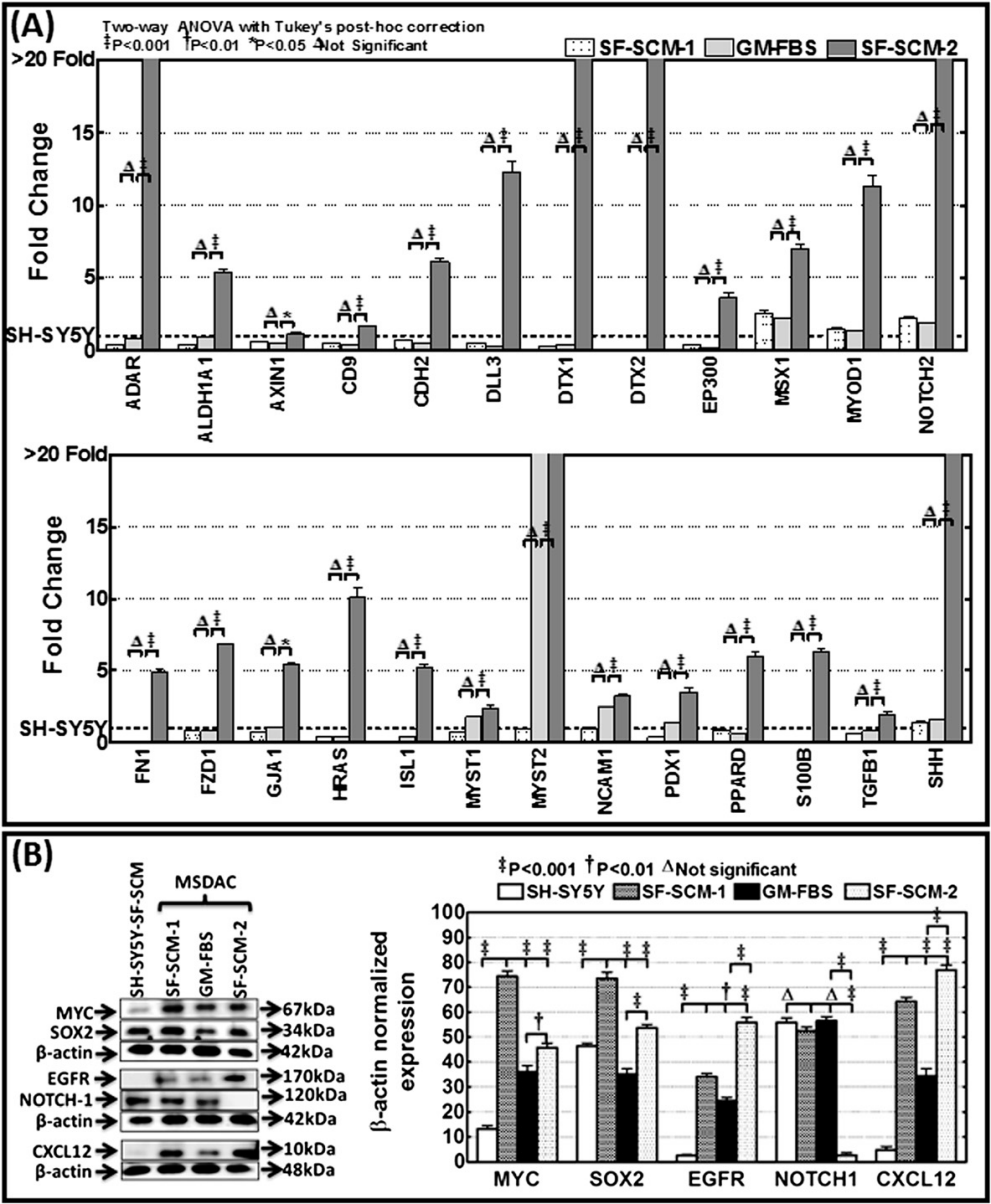
**Adaptive plasticity of stemness signaling in MSDACs cultured under alternated growth conditions. (A)** Histograms of QPCR profiling analysis showing stem cell-related transcriptional responses that were exceptionally activated with the regaining of the CSC phenotype under favorable niche conditions. These molecules did not show either loss or gain under the early CSC stage or the unfavorable and differentiated stage, but demonstrated a robust and significant activation when the MSDACs regained their CSC status, suggesting an intrinsic adaptive gain in stemness. **(B)** Representative blots showing the phenotypic conversion associated translational modifications in MYC, SOX2, EGFR, NOTCH1, and CXCL12. The phenotype-dependent modifications of these proteins corroborated well with their transcriptional expression data and validated the regain of the CSC status. CSC, cancer stem cell; MSDACs, metastatic site-derived aggressive cells.

To further substantiate our findings and underscore the associated alterations in stem cell-related signaling in this setting, we first investigated the plasticity-associated alterations in stem cell-related transcriptional machinery. We used a custom–made quantitative QPCR profile of stem cell-related molecules that govern EMT, self-renewal capacity, pluripotency maintenance, and drug resistance. Data mining of these altered transcriptional responses between the SCM-1(3G); SCM-1(3G) → GM-FBS(3G) and SCM-1(3G) → GM-FBS(3G) → SCM-2(3G) populations revealed three distinct clusters of genes including: (1) activation regained; (2) activation lost; and; (3) activation gained. Overall, the following 20 stem cell-related genes that were significantly activated in SCM-1(3G) were lost in SCM-1(3G) → GM-FBS(3G), and then regained in SCM-1(3G) → GM-FBS(3G) → SCM-2(3G): ASCL2, BGLAP, CDX2, COL1A1, COL2A1, CXCL12, DVL1, EGF2, EGFR1, FRAT, GJB1, GJB2, IGF1, KRT15, LIN28, PPARG, SIGMAR1, SNAI1, SNAI2, and SOX2 (Figure 5A). Reactivation of these molecules when the cells are reintroduced into the SF-SCM demonstrates the association of stem cell signaling with acquired plasticity in these MSDACs. However, the following 13 genes that were activated in SCM-1(3G) were completely lost in SCM-1(3G) → GM-FBS(3G) → SCM-2(3G): BMP2, BMP4, BTRC, CD4, CD44, CD8A, COL9A1, CTNNA1, DHH, DLL1, EGF4, GDF3 and TWIST1 (Figure 5B). In contrast with the other two conditions, 25 genes showed significant activation in SCM-1(3G) → GM-FBS(3G) → SCM-2(3G): ADAR, ALDH1A1, AXIN1, CD9, CDH2, DLL3, DTX1, DTX2, EP300, FN1, FZD1, GJA1, HRAS, ISL1, MSX1, MYOD1, NOTCH2, MYST1, MYST2, NCAM1, PDX1, PPARD, S100B, SHH, and TGFB1 (Figure 6A). Robust activation of these molecules in the MSDACs that were reintroduced to the SF-SCM demonstrates the ongoing acquisition of molecular rearrangements in MSDACs, and could drive the aggressive phenotype.

Immunoblotting analysis for selective proteins validated the observed transcriptional alterations and portrayed the translation of the response at the protein level. Compared with the parental SH-SY5Y cells, we observed a significant (*P* <0.001) activation of MYC, SOX2, EGFR, and CXCL12 in MSDACs (Figure 6B). This induced expression of MYC, SOX2, EGFR, and CXCL12 was lost in SF-SCM-1(3G) → GM-FBS(3G) MSDACs. However, the lost expression was significantly regained in SF-SCM-1(3G) → GM-FBS(3G) → SF-SCM-2(3G) MSDACs (Figure 6B). Expression of NOTCH1 was lost in the SF-SCM-1(3G) → GM-FBS(3G) → SF-SCM-2(3G) population. These results support the transcriptional data. Taken together, the transcriptional profiling and immunoblotting analysis demonstrate a significant association between the alterations of EMT, self-renewal capacity, pluripotency maintenance, and other stem cell-related signaling responses and the plasticity of the MSDACs. In addition, molecules that were only activated in the third phase suggested the continuous ongoing acquisition of stemness and drug resistance, which could explain the additional aggressiveness of this population.

## Discussion

Clinical outcomes for neuroblastoma vary greatly depending on stage and risk status. Patients with stage 1 and 2 disease are more likely to survive, as the disease will disappear with spontaneous maturation or regression, while patients with stage 4 and 4 s disease present with hematogenous metastasis, relapse/recurrence after therapy, and experience a dramatic decline in overall survival [22,31,47]. Studies worldwide, including those from our laboratory, focus on identifying the molecular drivers that switch favorable neuroblastoma to high-risk metastatic disease. Identifying these drivers could shift a huge archetype in the current treatment practice and benefit innumerable children with neuroblastoma.

Human cancers have been shown to contain populations of rapidly proliferating clonogens that can have a substantial impact on tumor control [14]. These tumor cells are responsible for failures in long-term remission and exhibit stem cell properties [15-21]. Conceptually, CSCs, a small subset of cells, constitute a reservoir of self-sustaining cells with the exclusive ability to self-renew and to spawn the heterogeneous lineages of cancer cells that comprise the tumor [48]. Emerging evidence from multiple tumor systems, including neuroblastoma, recognizes the driving role of such CSCs in tumor progression, relapse, recurrence, and poor clinical outcomes [49-51]. Researchers have postulated the unidirectional and bi-directional plasticity of these CSCs and highlighted their significance in tumor progression events [52,53]. In the current study, we examined the reversible and intrinsic adaptive plasticity of the human neuroblastoma MSDACs derived from clinically mimicking and translatable spontaneous high-risk metastatic tumors.

For the first time, the results of the present study demonstrate the reversible adaptive plasticity in aggressive neuroblastoma cells obtained from metastatic tumors. The MSDACs utilized for this study are well characterized and their cytogenetic, genetic, and molecular physiognomies are documented (data reported elsewhere). The results of the present study define their heightened survival characteristics, stem-like phenotype, and stem cell-related molecular blueprint. This is evident with the amplified expression of EMT, drug resistance, and pluripotency maintenance molecular response, as well as other stem cell-related transcriptional machinery (see Figure 2). These data validate the conceptual hypothesis that aggressive metastatic cells exhibit stem cell properties [15-21]. Utilizing validated human MSDACs that characteristically fit the CSC model to elucidate and typify plasticity allows us to closely relate these outcomes to clinical settings. Despite extensive neuroblastoma research recognizing the influence of CSCs on prognosis and clinical practices purging CSCs, our knowledge regarding CSC plasticity is limited. Chakrabarti and colleagues demonstrated the reversible plasticity of neuroblastoma cells [54] and further recognized the mechanism linking Id2 and TGFβ in drug-associated adaptive plasticity [55]. Consistently, our results demonstrated and validated the reversible plasticity in neuroblastoma cells. For the first time, this study explained neuroblastoma cells’ phenotype switching in MSDACs that typically fit the CSC model. Further, this study identified the intrinsic adaptiveness of the successive phenotype switching that clarifies the heterogeneity of aggressive neuroblastoma. This continuous ongoing acquisition of molecular rearrangements pertaining to stem cell-related signaling may drive the switch from favorable disease to aggressive neuroblastoma.

In addition to the reversible phenotypical changes exhibited by metastatic CSC-like cells, we observed the rescue of stem cell-related molecules. More importantly, we identified a cluster of activated molecules in the third phase (SF-SCM-1(3G) → GM-FBS(3G) → SF-SCM-2(3G)), consistent with adaptive plasticity. Identification of the stem cell-related molecular markers of SF-SEM-1(3G) and SF-SEM-2(3G) phenotypes allowed us to demonstrate that reversible adaptation is a definite process during tumor growth. The concept of CSC has gained support due to the indefinite tumorigenic potential, pluripotency maintenance, self-renewal capacity, and drug resistance of the proposed cells.

Although our results are consistent with this notion, showing a near-identical tumorosphere-forming capacity and stem cell-related molecular expression profiling, we also identified an added molecular response, demonstrating adaptive plasticity. As discussed above, this finding supports the hypothesis of cancer cell plasticity-associated tumor heterogeneity. Continuous ongoing acquisitions of molecular rearrangements in CSC seem to better enhance the plasticity and tumorigenic capacity of these cells.

The authors acknowledge the limitations of this study, including our analysis of pathway- focused stem cell-related transcriptional machinery rather than a whole genome approach, and that all experiments were confined to an *ex vivo* approach. However, these aggressive cells evade multimodal therapy and drive tumor progression in clinical settings while exhibiting CSC physiognomies. Further, stem cell-related molecular machinery has been shown to play a crucial role in phenotypic conversions. Hence, it is appropriate to identify stem cell machinery in neuroblastoma cells’ reversible and adaptive plasticity. The whole genome approach is beyond the scope of this manuscript. Investigation of the tumorigenic and metastatic potential of these cells under different phenotypes and the functional mechanism that instigates plasticity and high-risk aggressive disease are warranted and are currently underway in our laboratory with appropriate animal models.

## Conclusions

In conclusion, the results presented here for the first time show that aggressive MSDACs from high-risk metastatic disease fitting the CSC model are dynamically plastic and phenotypically respond to their microenvironment. These cells possess stem-like characteristics and are continuously acquiring molecular transitions that are needed to optimize their capacity to survive in a changing environment. It is evident that these cells inherit adaptive and additional molecular rearrangements that favor phenotype conversions on an as and when needed basis. Such a reversible and adaptive plasticity in highly metastatic CSC-like neuroblastoma cells may, hence, determine the clinical behavior and treatment response of neuroblastoma.

## Additional files

Additional file 1: Table S1. List of 93 stem-cell related genes, their Entrez gene name, cellular location, functional type and their known biomarker applications in the custom made Stemness/EMT transcriptome QPCR profiler examined in the current study. Additional file 2: Figure S1. Sequential images from a representative field obtained from high-content, time-lapse fluorescent imaging of DiI-stained MSDACs maintained in serum-free stem cell medium (SF-SCM-1). Cells were imaged in real time once every 20 minutes for an extended period of 18 hours using Operetta. MSDACs cultured in SF-SCM-1 showed significant cell proliferation and organized tumorosphere formation. Additional file 3: Figure S2. Sequential images obtained from time-lapse fluorescent imaging of MSDACs that were grown in SF-SCM-1 for three generations and subsequently maintained in growth medium supplemented with 10% FBS (GM-FBS) for an additional three generations. MSDACs cultured in SF-SCM-1 (3G) → GM-FBS(3G) showed consistent cell proliferation and monolayer spreading without any cellular aggregation or tumorosphere formation. Additional file 4: Figure S3. Sequential images obtained from time-lapse fluorescent imaging of MSDACs that were grown in SF-SCM-1(3G) → GM-FBS(3G) and subsequently reintroduced to the second phase of SF-SCM (SF-SCM-2) for an additional three generations. MSDACs cultured in SF-SCM-1 (3G) → GM-FBS(3G) → SF-SCM-2(3G) showed robust cell proliferation and consistent tumorosphere formation.

## Abbreviations

ANOVA: analysis of variance
Brd-U: bromodeoxyuridine
BSA: bovine serum albumin
CSCs: cancer stem cells
DAPI: 4',6-diamidino-2-phenylindole
DiI: 1,1′-dioctadecyl-3,3,3′,3′-tetramethylindocarbocyanine perchlorate
(D)MEM: (Dulbecco’s) modified Eagle’s medium
EGF: epidermal growth factor
EMT: epithelial-to-mesenchymal transition
GM-FBS: growth medium with fetal bovine serum
HR-NB: high-risk neuroblastoma
IF: immunofluorescence
iPSCs: induced pluripotent stem cells
miRNA: microRNA
MSDACs: metastatic site-derived aggressive cells
NB: neuroblastoma
NCSC: neural crest stem cell
PBS: phosphate-buffered saline
SF-SCM-1: serum-free stem cell medium
