# Erratum to: Metastatic neuroblastoma cancer stem cells exhibit flexible plasticity and adaptive stemness signaling

**DOI:** 10.1186/s13287-016-0291-6

**Published:** 2016-02-18

**Authors:** Vijayabaskar Pandian, Satishkumar Ramraj, Faizan H. Khan, Tasfia Azim, Natarajan Aravindan

**Affiliations:** Department of Radiation Oncology, University of Oklahoma Health Sciences Center, 940 Stanton L. Young Blvd., BMSB 737, Oklahoma City, OK 73104 USA

## Erratum

Due to a technical error during the production process, this article [1] was originally published with the incorrect citation *Stem Cell Res Ther* 2015, **6**:2. The citation has since been corrected, and the article should be cited as follows:

Pandian V**,** Ramraj S**,** Khan FH**,** Azim T and Aravindan N: **Metastatic neuroblastoma cancer stem cells exhibit flexible plasticity and adaptive stemness signaling.***Stem Cell Res Ther* 2015**, 6:**400.

The DOI of this article remains the same: doi: 10.1186/s13287-015-0002-8.

The Publisher is issuing this Erratum to alert readers in case they find versions or records of the article with the incorrect citation *Stem Cell Res Ther* 2015, **6**:2. We apologize for any inconvenience caused.
