# Supplementary material for: Metastatic neuroblastoma cancer stem cells exhibit flexible plasticity and adaptive stemness signaling
Source: Stem Cell Res Ther. 2015 Feb 20;6(1):2. doi: 10.1186/s13287-015-0002-8 (PMC4396071; doi:10.1186/s13287-015-0002-8)
Supplement: Additional file 1: Table S1. — List of 93 stem-cell related genes, their Entrez gene name, cellular location, functional type and their known biomarker applications in the custom made Stemness/EMT transcriptome QPCR profiler examined in the current study. [file 13287_2015_2_MOESM1_ESM.docx]

**Table 1.** List of 93 stem-cell related genes, their Entrez gene name, cellular location, functional type and their known biomarker applications in the custom made Stemness/EMT transcriptome QPCR profiler examined in the current study.

| Symbol | Entrez Gene Name | Location | Type(s) | Biomarker Application(s) |
| --- | --- | --- | --- | --- |
| ABCG2 | ATP-binding cassette, sub-family G (WHITE), member 2 | Plasma Membrane | transporter | efficacy,prognosis |
| ACAN | aggrecan | Extracellular Space | other | unspecified application |
| ADAR | adenosine deaminase, RNA-specific | Nucleus | enzyme |  |
| ALDH1A1 | aldehyde dehydrogenase 1 family, member A1 | Cytoplasm | enzyme | diagnosis,disease progression |
| ALDH2 | aldehyde dehydrogenase 2 family (mitochondrial) | Cytoplasm | enzyme | unspecified application |
| ALPI | alkaline phosphatase, intestinal | Plasma Membrane | phosphatase | unspecified application |
| APC | adenomatous polyposis coli | Nucleus | enzyme | diagnosis,disease progression,efficacy,prognosis,safety |
| ASCL2 | achaete-scute family bHLH transcription factor 2 | Nucleus | transcription regulator | diagnosis |
| AXIN1 | axin 1 | Cytoplasm | other |  |
| BGLAP | bone gamma-carboxyglutamate (gla) protein | Extracellular Space | other | diagnosis,efficacy,safety |
| BMP2 | bone morphogenetic protein 2 | Extracellular Space | growth factor |  |
| BMP3 | bone morphogenetic protein 3 | Extracellular Space | growth factor | diagnosis |
| BMP4 | bone morphogenetic protein 4 | Extracellular Space | growth factor |  |
| BTRC | beta-transducin repeat containing E3 ubiquitin protein ligase | Cytoplasm | enzyme |  |
| CCNA2 | cyclin A2 | Nucleus | other | efficacy,prognosis |
| CCND1 | cyclin D1 | Nucleus | transcription regulator | diagnosis,efficacy,prognosis,response to therapy,unspecified application |
| CCND2 | cyclin D2 | Nucleus | other | diagnosis,efficacy |
| CCNE1 | cyclin E1 | Nucleus | transcription regulator |  |
| CD4 | CD4 molecule | Plasma Membrane | transmembrane receptor | diagnosis,efficacy |
| CD44 | CD44 molecule (Indian blood group) | Plasma Membrane | enzyme | diagnosis,disease progression,prognosis,unspecified application |
| CD8A | CD8a molecule | Plasma Membrane | other | diagnosis |
| CD9 | CD9 molecule | Plasma Membrane | other | efficacy |
| CDC42 | cell division cycle 42 | Cytoplasm | enzyme |  |
| CDH1 | cadherin 1, type 1, E-cadherin (epithelial) | Plasma Membrane | other | diagnosis,disease progression,efficacy,prognosis |
| CDH2 | cadherin 2, type 1, N-cadherin (neuronal) | Plasma Membrane | other | diagnosis,prognosis |
| CDK1 | cyclin-dependent kinase 1 | Nucleus | kinase |  |
| CDX2 | caudal type homeobox 2 | Nucleus | transcription regulator | prognosis,unspecified application |
| COL1A1 | collagen, type I, alpha 1 | Extracellular Space | other | diagnosis |
| COL2A1 | collagen, type II, alpha 1 | Extracellular Space | other |  |

| Symbol | Entrez Gene Name | Location | Type(s) | Biomarker Application(s) |
| --- | --- | --- | --- | --- |
| COL9A1 | collagen, type IX, alpha 1 | Extracellular Space | other |  |
| CTNNA1 | catenin (cadherin-associated protein), alpha 1, 102kDa | Plasma Membrane | other | diagnosis |
| CXCL12 | chemokine (C-X-C motif) ligand 12 | Extracellular Space | cytokine | diagnosis,efficacy,prognosis,unspecified application |
| DHH | desert hedgehog | Extracellular Space | peptidase |  |
| DLL1 | delta-like 1 (Drosophila) | Plasma Membrane | enzyme |  |
| DLL3 | delta-like 3 (Drosophila) | Extracellular Space | other |  |
| DTX1 | deltex 1, E3 ubiquitin ligase | Nucleus | transcription regulator |  |
| DTX2 | deltex 2, E3 ubiquitin ligase | Nucleus | other |  |
| DVL1 | dishevelled segment polarity protein 1 | Cytoplasm | other |  |
| EP300 | E1A binding protein p300 | Nucleus | transcription regulator |  |
| FGF1 | fibroblast growth factor 1 (acidic) | Extracellular Space | growth factor | prognosis |
| FGF2 | fibroblast growth factor 2 (basic) | Extracellular Space | growth factor | diagnosis,efficacy,prognosis |
| FGF3 | fibroblast growth factor 3 | Extracellular Space | growth factor |  |
| FGF4 | fibroblast growth factor 4 | Extracellular Space | growth factor |  |
| FGFR1 | fibroblast growth factor receptor 1 | Plasma Membrane | kinase | diagnosis,prognosis,unspecified application |
| FN1 | fibronectin 1 | Extracellular Space | enzyme | diagnosis,efficacy,prognosis,unspecified application |
| FOXA2 | forkhead box A2 | Nucleus | transcription regulator | disease progression |
| FRAT1 | frequently rearranged in advanced T-cell lymphomas 1 | Cytoplasm | other |  |
| FZD1 | frizzled class receptor 1 | Plasma Membrane | G-protein coupled receptor |  |
| GDF3 | growth differentiation factor 3 | Extracellular Space | growth factor |  |
| GJA1 | gap junction protein, alpha 1, 43kDa | Plasma Membrane | transporter |  |
| GJB1 | gap junction protein, beta 1, 32kDa | Plasma Membrane | transporter | unspecified application |
| GJB2 | gap junction protein, beta 2, 26kDa | Plasma Membrane | transporter | disease progression |
| GSK3B | glycogen synthase kinase 3 beta | Nucleus | kinase | efficacy |
| HDAC2 | histone deacetylase 2 | Nucleus | transcription regulator |  |
| HRAS | Harvey rat sarcoma viral oncogene homolog | Plasma Membrane | enzyme | prognosis,safety |
| HSPA9 | heat shock 70kDa protein 9 (mortalin) | Cytoplasm | other |  |
| IGF1 | insulin-like growth factor 1 (somatomedin C) | Extracellular Space | growth factor | diagnosis,efficacy,prognosis,safety |
| ISL1 | ISL LIM homeobox 1 | Nucleus | transcription regulator |  |
| JAG1 | jagged 1 | Extracellular Space | growth factor | efficacy |
| KAT7 | K(lysine) acetyltransferase 7 | Nucleus | enzyme |  |
| KAT8 | K(lysine) acetyltransferase 8 | Nucleus | enzyme |  |
| KLF5 | Kruppel-like factor 5 (intestinal) | Nucleus | transcription regulator |  |

| Symbol | Entrez Gene Name | | Location | | Type(s) | | Biomarker Application(s) |
| --- | --- | --- | --- | --- | --- | --- | --- |
| KRT15 | keratin 15 | Cytoplasm | | other | |  | |
| LIN28A | lin-28 homolog A (C. elegans) | Cytoplasm | | other | |  | |
| MME | membrane metallo-endopeptidase | Plasma Membrane | | peptidase | | diagnosis,efficacy,unspecified application | |
| MSX1 | msh homeobox 1 | Nucleus | | transcription regulator | |  | |
| MYC | v-myc avian myelocytomatosis viral oncogene homolog | Nucleus | | transcription regulator | | diagnosis,efficacy,prognosis,response to therapy,unspecified application | |
| MYOD1 | myogenic differentiation 1 | Nucleus | | transcription regulator | | prognosis | |
| NCAM1 | neural cell adhesion molecule 1 | Plasma Membrane | | other | | efficacy,prognosis | |
| NEUROG2 | neurogenin 2 | Nucleus | | other | |  | |
| NOTCH1 | notch 1 | Plasma Membrane | | transcription regulator | | diagnosis,efficacy | |
| NOTCH2 | notch 2 | Plasma Membrane | | transcription regulator | | unspecified application | |
| NUMB | numb homolog (Drosophila) | Plasma Membrane | | other | | efficacy | |
| OCLN | occludin | Plasma Membrane | | enzyme | |  | |
| PARD6A | par-6 family cell polarity regulator alpha | Plasma Membrane | | other | |  | |
| PDX1 | pancreatic and duodenal homeobox 1 | Nucleus | | transcription regulator | |  | |
| PPARD | peroxisome proliferator-activated receptor delta | Nucleus | | ligand-dependent nuclear receptor | | diagnosis | |
| PPARG | peroxisome proliferator-activated receptor gamma | Nucleus | | ligand-dependent nuclear receptor | | diagnosis,efficacy,unspecified application | |
| RB1 | retinoblastoma 1 | Nucleus | | transcription regulator | | diagnosis,efficacy,prognosis,unspecified | |
| S100B | S100 calcium binding protein B | Cytoplasm | | other | | diagnosis,efficacy | |
| SHH | sonic hedgehog | Extracellular Space | | peptidase | | diagnosis,efficacy,response to therapy,unspecified application | |
| SIGMAR1 | sigma non-opioid intracellular receptor 1 | Plasma Membrane | | G-protein coupled receptor | |  | |
| SNAI1 | snail family zinc finger 1 | Nucleus | | transcription regulator | | diagnosis,efficacy | |
| SNAI2 | snail family zinc finger 2 | Nucleus | | transcription regulator | |  | |
| SOX1 | SRY (sex determining region Y)-box 1 | Nucleus | | transcription regulator | |  | |
| SOX2 | SRY (sex determining region Y)-box 2 | Nucleus | | transcription regulator | | diagnosis,prognosis | |
| T | T, brachyury homolog (mouse) | Nucleus | | transcription regulator | |  | |
| TBX2 | T-box 2 | Nucleus | | transcription regulator | |  | |
| TERT | telomerase reverse transcriptase | Nucleus | | enzyme | | diagnosis,disease progression,prognosis | |
| TGFB1 | transforming growth factor, beta 1 | Extracellular Space | | growth factor | | diagnosis,efficacy,prognosis,safety,unspecified | |
| TUBB3 | tubulin, beta 3 class III | Cytoplasm | | other | | response to therapy | |
| TWIST1 | twist family bHLH transcription factor 1 | Nucleus | | transcription regulator | | efficacy | |
| TWIST2 | twist family bHLH transcription factor 2 | Nucleus | | transcription regulator | |  | |
