# Supplementary material for: Metastatic neuroblastoma cancer stem cells exhibit flexible plasticity and adaptive stemness signaling
Source: Stem Cell Res Ther. 2015 Feb 20;6(1):2. doi: 10.1186/s13287-015-0002-8 (PMC4396071; doi:10.1186/s13287-015-0002-8)
Supplement: Additional file 2: Figure S1. — Sequential images from a representative field obtained from high-content, time-lapse fluorescent imaging of DiI-stained MSDACs maintained in serum-free stem cell medium (SF-SCM-1). Cells were imaged in real time once every 20 minutes for an extended period of 18 hours using Operetta. MSDACs cultured in SF-SCM-1 showed significant cell proliferation and organized tumorosphere formation. [file 13287_2015_2_MOESM2_ESM.pptx]

## Slide 1
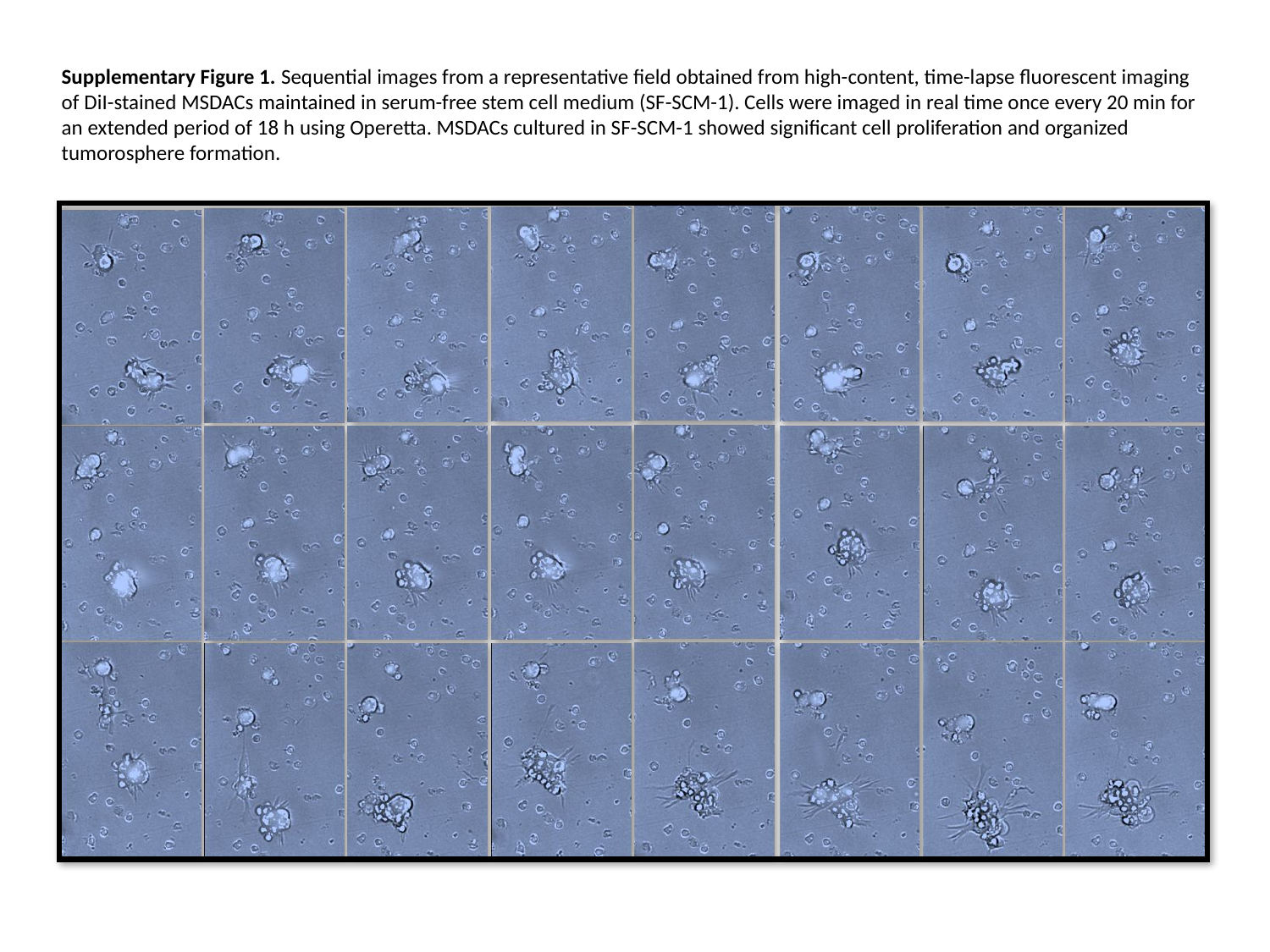

Supplementary Figure 1. Sequential images from a representative field obtained from high-content, time-lapse fluorescent imaging of DiI-stained MSDACs maintained in serum-free stem cell medium (SF-SCM-1). Cells were imaged in real time once every 20 min for an extended period of 18 h using Operetta. MSDACs cultured in SF-SCM-1 showed significant cell proliferation and organized tumorosphere formation.
