# Supplementary material for: Metastatic neuroblastoma cancer stem cells exhibit flexible plasticity and adaptive stemness signaling
Source: Stem Cell Res Ther. 2015 Feb 20;6(1):2. doi: 10.1186/s13287-015-0002-8 (PMC4396071; doi:10.1186/s13287-015-0002-8)
Supplement: Additional file 3: Figure S2. — Sequential images obtained from time-lapse fluorescent imaging of MSDACs that were grown in SF-SCM-1 for three generations and subsequently maintained in growth medium supplemented with 10% FBS (GM-FBS) for an additional three generations. MSDACs cultured in SF-SCM-1 (3G) → GM-FBS(3G) showed consistent cell proliferation and monolayer spreading without any cellular aggregation or tumorosphere formation. [file 13287_2015_2_MOESM3_ESM.pptx]

## Slide 1
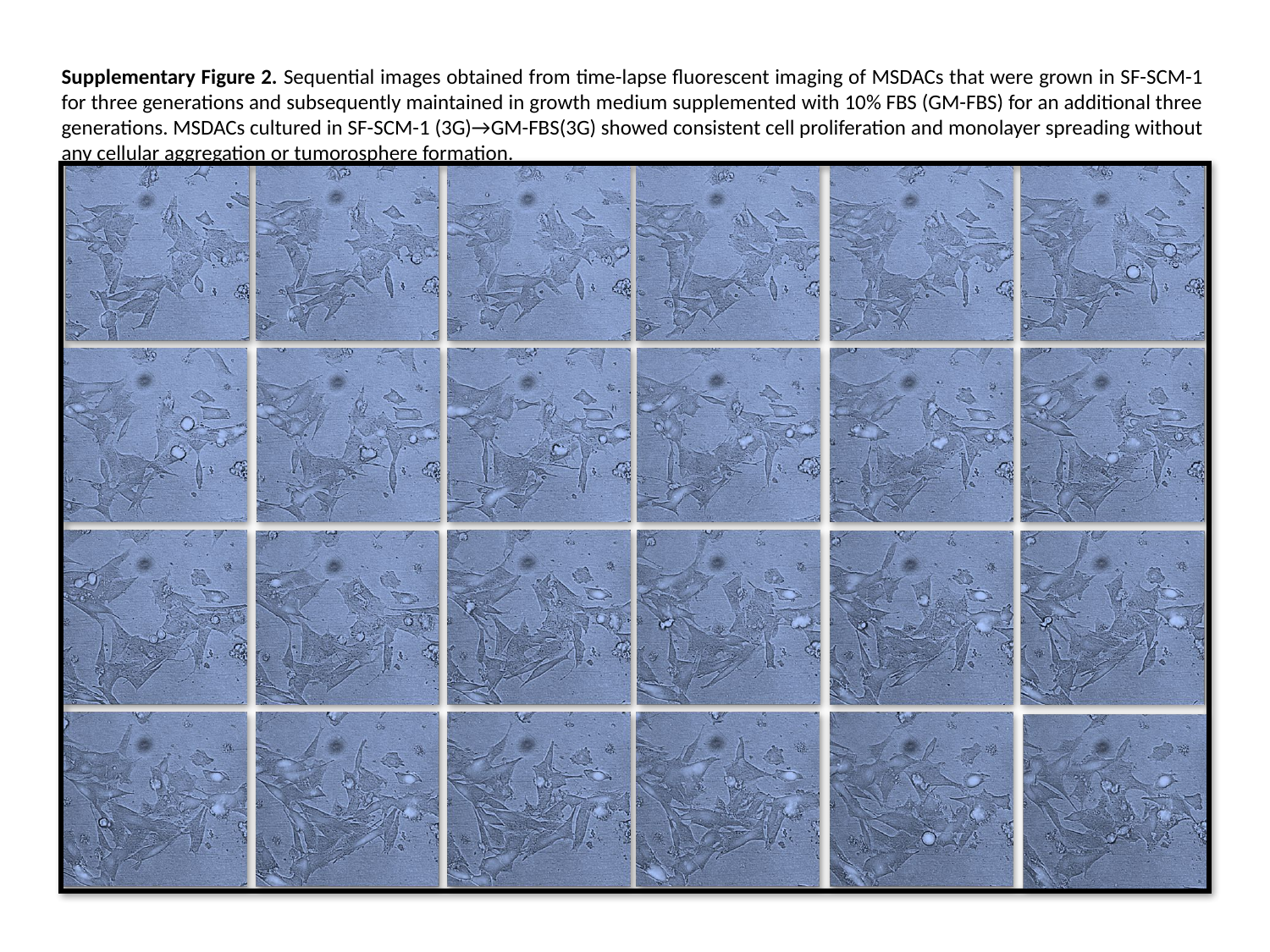

Supplementary Figure 2. Sequential images obtained from time-lapse fluorescent imaging of MSDACs that were grown in SF-SCM-1 for three generations and subsequently maintained in growth medium supplemented with 10% FBS (GM-FBS) for an additional three generations. MSDACs cultured in SF-SCM-1 (3G)→GM-FBS(3G) showed consistent cell proliferation and monolayer spreading without any cellular aggregation or tumorosphere formation.
