# Supplementary material for: Metastatic neuroblastoma cancer stem cells exhibit flexible plasticity and adaptive stemness signaling
Source: Stem Cell Res Ther. 2015 Feb 20;6(1):2. doi: 10.1186/s13287-015-0002-8 (PMC4396071; doi:10.1186/s13287-015-0002-8)
Supplement: Additional file 4: Figure S3. — Sequential images obtained from time-lapse fluorescent imaging of MSDACs that were grown in SF-SCM-1(3G) → GM-FBS(3G) and subsequently reintroduced to the second phase of SF-SCM (SF-SCM-2) for an additional three generations. MSDACs cultured in SF-SCM-1 (3G) → GM-FBS(3G) → SF-SCM-2(3G) showed robust cell proliferation and consistent tumorosphere formation. [file 13287_2015_2_MOESM4_ESM.pptx]

## Slide 1
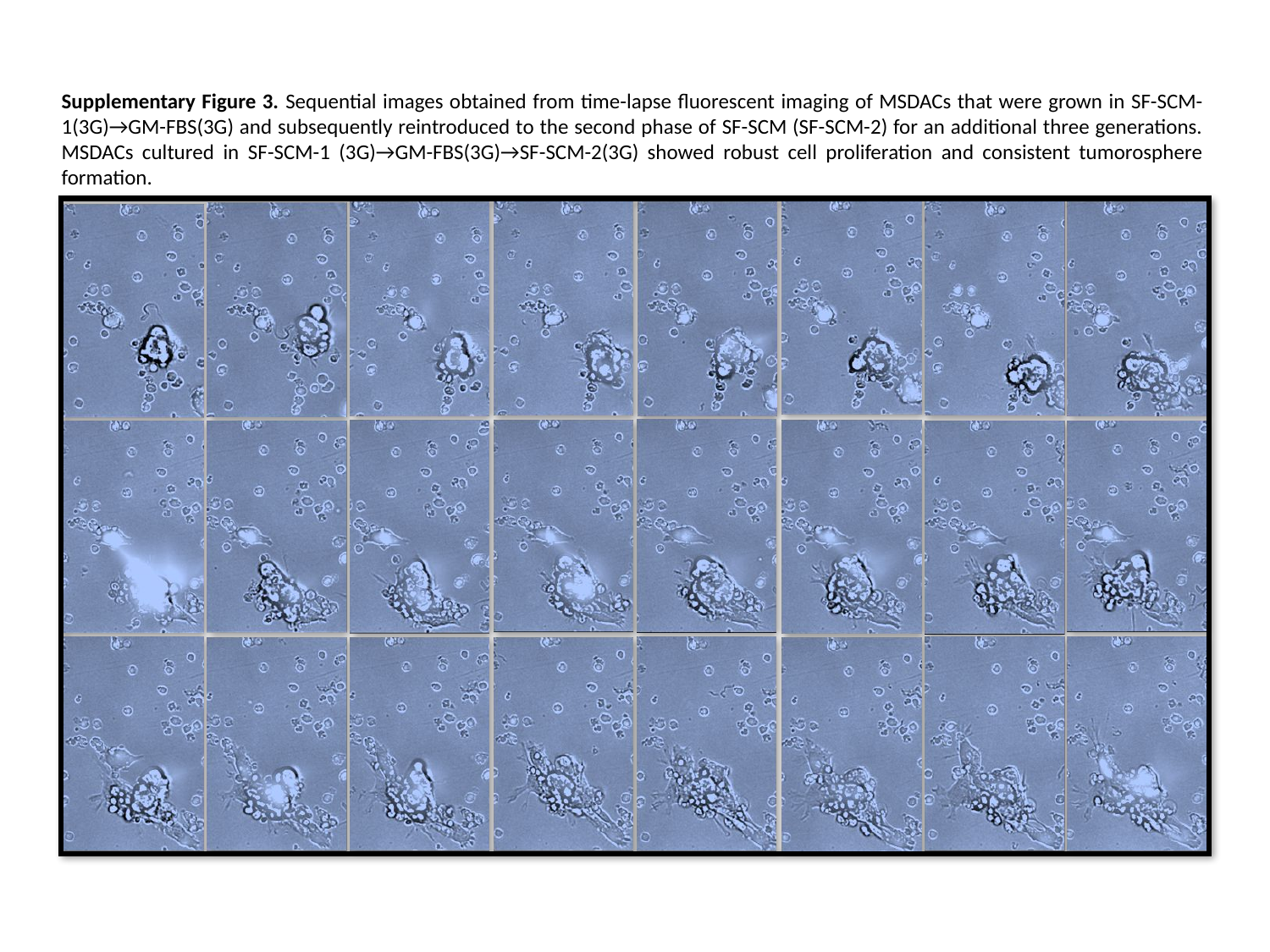

Supplementary Figure 3. Sequential images obtained from time-lapse fluorescent imaging of MSDACs that were grown in SF-SCM-1(3G)→GM-FBS(3G) and subsequently reintroduced to the second phase of SF-SCM (SF-SCM-2) for an additional three generations. MSDACs cultured in SF-SCM-1 (3G)→GM-FBS(3G)→SF-SCM-2(3G) showed robust cell proliferation and consistent tumorosphere formation.
